# Roles of Non-Structural Protein 4A in Flavivirus Infection

**DOI:** 10.3390/v13102077

**Published:** 2021-10-15

**Authors:** Paeka Klaitong, Duncan R. Smith

**Affiliations:** Institute of Molecular Biosciences, Mahidol University, Nakhon Pathom 73170, Thailand; paeka_p@hotmail.com

**Keywords:** flavivirus, transmembrane protein, autophagy, congenital Zika syndrome, interferon response, unfolded protein response

## Abstract

Infections with viruses in the genus *Flavivirus* are a worldwide public health problem. These enveloped, positive sense single stranded RNA viruses use a small complement of only 10 encoded proteins and the RNA genome itself to remodel host cells to achieve conditions favoring viral replication. A consequence of the limited viral armamentarium is that each protein exerts multiple cellular effects, in addition to any direct role in viral replication. The viruses encode four non-structural (NS) small transmembrane proteins (NS2A, NS2B, NS4A and NS4B) which collectively remain rather poorly characterized. NS4A is a 16kDa membrane associated protein and recent studies have shown that this protein plays multiple roles, including in membrane remodeling, antagonism of the host cell interferon response, and in the induction of autophagy, in addition to playing a role in viral replication. Perhaps most importantly, NS4A has been implicated as playing a critical role in fetal developmental defects seen as a consequence of Zika virus infection during pregnancy. This review provides a comprehensive overview of the multiple roles of this small but pivotal protein in mediating the pathobiology of flaviviral infections.

## 1. Introduction

The genus *Flavivirus* of the family *Flaviviridae* comprises over 50 species of arthropod-borne enveloped viruses [1]. Most viruses in this genus are transmitted to vertebrate hosts through the bite of infected hematophagous arthropods, although some have arthropod- or vertebrate-restricted transmission cycles [2]. Around half of the viruses assigned to a species in the genus *Flavivirus* are known human pathogens [3], of which the medically important viruses causing public health problems worldwide are dengue virus (DENV: DENV 1 to DENV 4), Zika virus (ZIKV), Japanese encephalitis virus (JEV), West Nile virus (WNV) and yellow fever virus (YFV), which are transmitted by mosquitoes, and tick-borne encephalitis virus (TBEV) which is transmitted by ticks. Flavivirus infection of humans causes a variety of manifestations ranging from no symptoms or non-severe flu-like symptoms to severe or even lethal symptoms such as hemorrhagic fever and shock syndrome for DENV infection, Guillain–Barré syndrome and fetal microcephaly for ZIKV infection, meningitis and encephalitis for JEV, WNV, TBEV infections and liver failure and jaundice for YFV infection. Vaccination is considered a reasonable method to prevent flavivirus infections. Vaccines for JEV, YFV and TBEV are currently licensed for use in humans [4], and while a vaccine for DENV is licensed in some countries, the occurrence of more severe disease in some vaccinated individuals [5] has served to limit its application. However, vaccines for the other human pathogenic flaviviruses are either unable to elicit broadly protective immune responses or are in varying stages of development. In addition, to date, there is no specific antiviral drug available to treat any flaviviral infection. Viral components offer potential drug targets, but as flaviviruses mutate rapidly due to their low-fidelity replication process, drug-resistant strains can rapidly emerge. Host-oriented therapeutics have been therefore considered as a potential alternative.

For a successful infection, viruses must manipulate the host cellular environment to establish an optimal platform for their genome replication, protein production, and virion assembly and egress. To this end, viruses utilize both viral and host factors to aid in reorganization of intracellular membranes, manipulation of host signaling and metabolic pathways, and evasion of host immune responses.

Flaviviruses possess a non-segmented positive-sense single-stranded RNA genome of 10–11 kb. The genome is modified at the 5′ end with a m7GpppAm cap structure and lacks a poly-A tail. The genome contains a long open reading frame (ORF) flanked by a 5′ untranslated region (UTR) and a 3′ UTR of approximately 100 and 400–700 nucleotides, respectively [6]. The 5′and 3′ UTR usually form highly conserved secondary and tertiary structures essential for RNA replication and protein translation [7]. The genome is translated in close association with intracellular membranes, giving rise to a single polyprotein precursor. The structural proteins (capsid, precursor membrane (prM) and envelope (E)) encompass the 5′ one-fourth of the polyprotein, while the non-structural (NS) proteins (NS1, NS2A, NS2B, NS3, NS4A, NS4B and NS5) occupy the remainder of the polyprotein. The polyprotein is co- and post-translationally processed into individual components by cellular proteases and the viral NS2B–NS3 protease complex. While the structural proteins comprise the virion, the NS proteins are primarily responsible for viral RNA replication, virion assembly and modulation of host immune responses [8,9,10,11,12,13,14].

The role of flavivirus NS1 in viral replication is not fully understood, but it has been shown to function at a very early stage in viral RNA replication [15,16,17,18]. NS1 has also been shown to have a role in the modulation of host innate immune response [19,20,21], and in viral neuroinvasiveness [22]. NS2A and NS4B have been suggested to be involved in anchoring the viral replication complexes to cellular membranes [23] and to act as interferon (IFN) antagonists by blocking IFN-α/β signaling [12]. Furthermore, NS2A is likely to play an important role in coordinating the shift between RNA replication and RNA packaging processes [6,24] and to be involved in virion assembly [25,26]. NS2B forms a stable complex with NS3 and acts as a cofactor for the NS2B-NS3 serine protease [27], which is responsible for viral polyprotein processing. In addition to the protease activity, NS3 also possesses RNA-stimulated nucleoside triphosphatase (NTPase), RNA triphosphatase and RNA helicase activity essential for viral RNA replication and capping [28,29]. NS5 contains an RNA-dependent RNA polymerase (RdRp) activity responsible for viral RNA replication, and a methyltransferase activity involved in capping of the newly synthesized RNA genome [30,31]. Thus far, however, the functions of NS4A in flavivirus infection remains poorly characterized. The aim of this review is to draw together the knowledge regarding the roles of NS4A in flavivirus infection, and to shed light on the design and development of antiviral therapeutics.

## 2. Roles of NS4A in Flavivirus Replication

### 2.1. NS4A Mediates Flavivirus-Induced Membrane Remodeling

Several studies have described ultrastructural changes in cellular membranes, especially in the perinuclear region of cells infected with flaviviruses. In general, the earliest event is the extensive proliferation of endoplasmic reticulum (ER) membranes, followed by the formation of double-membrane vesicles called vesicular packets (VPs) inside the ER lumen [32,33,34,35]. The appearance of paracrystalline arrays (PCs) or convoluted membranes (CMs) contiguous with the ER has been described for WNV infection [36,37]. VPs are the sites of viral RNA replication as they contain double-stranded RNA (dsRNA) and the viral NS5 RdRp [17,35,37,38]. CMs have been suggested to be the sites of proteolytic cleavage of the viral polyprotein as evidenced by the fact that these structures are colocalized with the viral NS2B and NS3 (comprising the viral protease) [39]. The flavivirus-induced membrane reorganization is therefore thought to give rise to proximal, yet distinct, specialized scaffolds for viral RNA replication versus viral protein translation and processing [40]. However, the exact mechanism underlying these virus-induced membrane reorganizations remains unclear.

**Figure 1:**
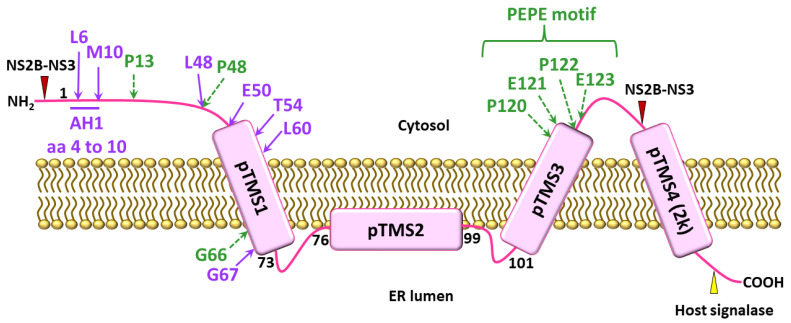
A membrane topology model of flavivirus NS4As. NS4A consists of an N-terminal cytosolic region and four predicted transmembrane segments: pTMS1, 2, 3 and 4 with the latter referred to as the 2k fragment. The red and yellow triangles mark the site of viral NS2B-NS3 protease and host signalase cleavage sites, respectively. Thick purple and dashed green arrows indicate specific amino acid (aa) residues in DENV and WNV NS4A, respectively, contributing to viral replication. AH1 is the experimentally determined amphipathic helix 1 of DENV NS4A. The position of critical amino acids is indicated.

**Figure 2:**
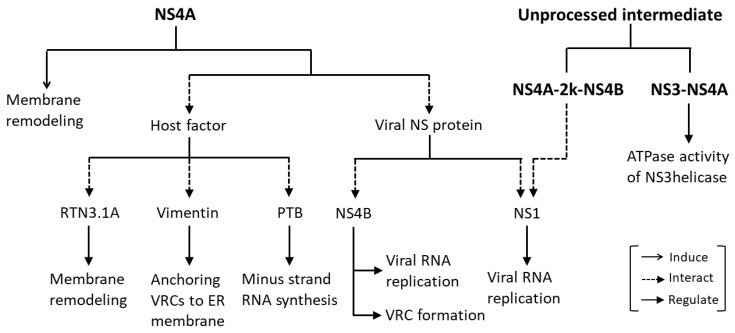
Roles of NS4As in flavivirus replication. NS4As induce membrane remodeling similar to that induced by flavivirus infection. NS4A and the unprocessed intermediates containing NS4A are essential components of viral replication complexes (VRCs), that interacts with host factors or other flavivirus NS proteins to promote efficient viral replication.

NS4A is a 16-kDa transmembrane ER resident protein consisting of an N-terminal cytosolic region and four predicted transmembrane segments (pTMSs) [41,42]. The domains pTMS1 and pTMS3 span the ER membrane, while pTMS2 is embedded in the luminal leaflet of the ER membrane. The C-terminal pTMS4, referred to as the 2k fragment, spans the ER membrane (Figure 1) and acts as a signal peptide for the ER localization of NS4B. The 2k fragment is removed from mature NS4A by the NS2B-NS3 protease [41,43]. It has been shown that all four pTMSs of DENV NS4A possess membrane targeting capabilities and are able to mediate membrane association when expressed independently [41]. NS4A has been shown to play a major role in the flavivirus-induced membrane remodeling (Figure 2). Heterologous expression of WNV NS4A retaining the 2k fragment induced cytoplasmic membrane remodeling, resembling those events observed upon WNV infection. Removal of the 2k fragment on the other hand, impaired the ability of WNV NS4A to induce membrane remodeling, and resulted in the redistribution of this protein to the Golgi apparatus [44]. In contrast to the WNV NS4A, proteolytic removal of the 2k fragment appears to be necessary for heterologously expressed DENV NS4A to induce ER membrane remodeling which is similar to that induced by DENV infection [41]. These findings suggest that 2k regulates the function of NS4A in modulating cellular membranes through distinct mechanisms in different flaviviruses.

The N-terminus of WNV NS4A has been shown to contribute to the stability of the protein, which is essential for facilitating efficient WNV replication. Mutations at P13, P48, D49 and G66 showed variable defects in viral replication and membrane remodeling (Figure 1, Table 1), with the mutations P13A and D49A causing lethal and mild defects, respectively. The highly attenuated mutations P48A and G66A coincidingly showed an increase in a specific proteasome-mediated degradation of WNV NS4A, leading to a substantial reduction in membrane proliferation, in particular the proliferation of CM and PC structures, and eventually resulting in inefficient viral replication [45]. The residues P13, P48 and G66 of NS4As have been shown to be highly conserved within WNV species, and between members in the genus *Flavivirus* [46,47]; thus, these residues are highly likely to contribute to the protein stability.

It is still unknown how NS4A contributes to the substantial alterations of cellular membranes, nevertheless, several mechanisms have been proposed [48,49]. Insertion of amphipathic α-helix (AH) into one leaflet of membrane bilayers, as well as oligomerization of membrane proteins in or above the polar lipid–water interface are among the mechanisms suggested to promote the induction of membrane curvature [48]. The closely related hepatitis C virus (HCV; genus *Hepacivirus*, family *Flaviviridae*) NS4B contains an N-terminal AH that is able to induce membrane alterations when expressed independently [50]. Homo-oligomerization of HCV NS4B has been reported, and is likely to be required for the induction of membrane alterations [51]. A speculative mechanism to account for the cellular membrane alterations induced by HCV NS4B is that NS4B induces membrane curvature by inserting its AHs into the membranes, and then homo-oligomerization makes large NS4B complexes that force membrane curvature [52].

The cytosolic N-terminal region (amino acids 1 to 48) of DENV NS4A has been analyzed and found to contain two experimentally determined AHs (AH1: amino acids 4 to 10 (Figure 1, Table 1); AH2: amino acids 15 to 31) that are separated by an unstructured linker [53]. The DENV NS4A (1 to 48) has been shown to bind tightly to membrane bilayers, particularly to the negatively charged bilayer [42,53,54]. Disruption of the amphipathic character of AH1 by L6E; M10E mutations reduced the membrane binding of the DENV NS4A (1 to 48) [42,53], indicating that AH1 has a high affinity for membranes. DENV NS4A has also been shown to form homo-oligomers in infected cells or when expressed independently [42,55]. The AH1 was found to have a significant contribution to the homo-oligomerization of DENV NS4A, as the L6E; M10E mutations in the AH1 reduced homo-oligomerization (Figure 1, Table 1), but did not affect its localization [42]. The pTMS1 (amino acids 50 to 76) has also been shown to be a major determinant for homo-oligomerization of DENV 2 NS4A. Specifically, pTMS1 alone exhibited homo-oligomerization activity comparable to that of full-length NS4A, while the cytosolic N-terminal region (amino acids 1 to 50) retained only 20% of the full length NS4A homo-oligomerization activity. Single point mutations E50A and G67A in the pTMS1 decreased homo-oligomerization and stability of DENV 2 NS4A [55] (Figure 1, Table 1). 

While a peptide (amino acids 1 to 48) of ZIKV NS4A encompassing the entire cytosolic N-terminal region has been found to form a random coil in solution [56], the peptide (amino acids 4 to 58) spanning most of the cytosolic N-terminal region and a third of the pTMS1 has been found to be partially folded in solution [57]. The peptide (amino acids 4 to 58) was found to contain two predicted AHs (AH1: amino acids 15 to 33; AH2: amino acids 38 to 55) and shown to bind membranes, as its helical contents were increased in the presence of liposomes [57], consistent with previous reports for DENV NS4A [42,53,54]. Moreover, the peptide (amino acids 4 to 58) was also found to form homotrimers even in the absence of detergents or lipid membranes, suggesting that this part of the protein is essential for homo-oligomerization of ZIKV NS4A [57] (Table 1). 

All of these findings suggest that induction of cellular membrane remodeling by flavivirus NS4As might be mediated by a mechanism similar to that used by HCV NS4B. Nevertheless, the predicted membrane topology of DENV NS4A has suggested that the N-terminal 49 residues (containing AHs [53]) are exposed to the cytosol [41]. In addition, for a number of viral membrane-bound proteins involved in viral replication such as the NS4A and NS5A proteins of HCV, GB virus and bovine viral diarrhea virus which contain AHs [58,59,60], the AHs have been shown to play a role in mediating membrane association of these viral proteins [59,61]. Therefore, flavivirus NS4As are more likely to induce membrane curvature by associating their cytosolic N-terminal AHs to the membranes as opposed to inserting them directly into the membranes.

**Table 1:** Contributions of specific amino acid residues within flavivirus NS4As to viral replication.

NS4A	Amino Acid Residue	Contribution	Reference
DENV NS4A	L6 and M10 within AH1: aa 4 to 10	Membrane binding, homo-oligomerization and viral replication	[55]
	E50 and G67	Homo-oligomerization, protein stability and viral replication	
	aa 1 to 50	Vimentin interaction to mediate the anchoring of VRCs to ER membrane	[62]
	L48, T54 and L60	NS4A-NS4B interaction and viral replication	[63]
WNV NS4A	P13	Viral replication	[45]
	P48 and G66	Protein stability	
	Potential CRAC motif: ^25^L-(X)-^29^Y-(X)-^36^K	Membrane remodeling, promoting VRC assembly at cholesterol-rich microdomains within the ER membrane	[47]
	P120-E121-P122-E123 (PEPE motif)	VRC formation and promoting the cleavage of 2k from NS4A	[46]
	aa 1 to 50	Regulating ATPase activity of NS3 helicase	[64]
ZIKV NS4A	aa 4 to 58 (containing AH1: aa 15 to 33; AH2: aa 38 to 55)	Membrane binding and homo-oligomerization	[57]

Homo-oligomerization of DENV NS4A has been demonstrated to have a biological importance in viral replication. The reductions in homo-oligomerization of DENV 2 NS4A caused by either the L6E, M10E mutations in AH1 or the E50A and G67A mutations in pTMS1, lead to attenuated viral replication [42,55] (Figure 1, Table 1), which was thought to result from the decreased NS4A protein stability as a consequence of weakened NS4A homo-oligomerization [55].

The reticulon (RTN) protein family is a group of membrane-bound proteins that are primarily involved in promoting membrane curvature and vesicle formation [65,66]. Upon WNV, DENV and ZIKV infection, RTN3.1A has been found to be recruited to the virus-induced modified ER membranes comprising viral replication complexes. RTN3.1A was shown to interact with WNV NS4A potentially, through the pTMSs at the N-terminus of NS4A. Knockdown of *RTN3.1A* not only reduced WNV, DENV, and ZIKV replication, but also promoted degradation of viral proteins particularly, NS4A, with the proteasome in part contributing to the viral protein degradation. In addition, silencing of *RTN3.1A* affected virus-induced membrane remodeling; specifically, the numbers and/or sizes of CMs, PCs and VPs were reduced, or VPs were aberrantly elongated, coinciding with an increase in the number of immature virus particles. These findings suggests that RTN3.1A stabilizes NS4A and functions cooperatively with the membrane-remodeling capability of NS4A to facilitate virus-induced membrane remodeling for efficient flavivirus replication [67] (Figure 2).

### 2.2. NS4A Is an Essential Component of the Viral Replication Complex

Viral RNA replication takes place in viral replication complexes (VRCs) situated in VPs. VRCs are composed of viral dsRNA, viral proteins and essential host factors [52]. Although, the exact composition of the VRCs is still unknown, all flaviviral NS proteins including NS4A have been suggested to be components of the VRCs, as co-localization of NS proteins with viral dsRNA in VPs, and interactions among the NS proteins have been identified [37,39,55,68,69,70,71,72].

WNV replication has been found to take place in close association with cholesterol-rich microdomains within the ER membrane [47]. WNV NS4A has been found to contain a potential cholesterol recognition/interaction amino acid consensus (CRAC) motif (L/V^24^-X_(1–5)_-Y^28^-X_(1__–5__)_-R/K^35^) which is a ^25^L-(X)-^29^Y-(X)-^36^K motif, near its N-terminus [47]. This motif is highly conserved among members of the Japanese encephalitis subgroup but shows low degrees of sequence similarities with the other members in the genus *Flavivirus*. Mutant viruses harboring either mutation of Y/S at position 28 or K/L at position 35 or double mutation Y/S + K/L showed varying degrees of attenuated phenotypes, with the virus harboring the double mutation Y/S + K/L being extremely attenuated followed by the viruses harboring single mutation Y/S and K/L, respectively. These results suggest that the CRAC motif within the WNV NS4A plays a significant role in facilitating efficient viral replication. Importantly, the Y/S mutation was shown to significantly impair the ability of the mutant virus to recruit viral components and cellular factors known to localize to the VRCs [73] to effectively form VRCs at the cholesterol-rich microdomains within the ER membrane [47]. Moreover, virus harboring the Y/S mutation was also found to be defective in induction of the CM/PC structure [47]. Collectively, these findings suggest that the CRAC motif within the N-terminus of the WNV NS4A plays a significant role in promoting cytoplasmic membrane remodeling and VRC assembly to specific cholesterol-rich microdomains within the ER membrane, thus facilitating efficient virus replication. However, whether the CRAC motif within WNV NS4A confers direct binding with cellular chloresterol remains inconclusive [47].

A conserved Pro–Glu–Pro–Glu (PEPE) motif in the hydrophobic C-terminus of WNV NS4A has been shown to be essential for VRC formation (Figure 1, Table 1). Mutations in the PEPE motif impaired VRC formation which in turn abolished viral RNA replication and virion production. The PEPE motif was also found to contribute to proteolytic cleavage to remove the 2k fragment from WNV NS4A, as shown by the fact that the mutations in the PEPE motif perturbed proteolytic processing at the NS2B-NS3 cleavage site upstream of the 2k region, specifically at the first proline and downstream glutamic acid residues [46]. The authors of that study were inclined to believe that as the PEPE motif was in close proximity to NS2B-NS3 cleavage site, the mutations were preventing NS2B-NS3 protease accessibility and thus activity, resulting in incorrect processing of NS4A which impeded VRC formation [46].

Upon DENV infection, vimentin, a component of intermediate filaments, is redistributed to the perinuclear site, where it shows co-localization with DENV-induced ER-derived membranous compartments, and with NS4A representing VRCs. Gene silencing of vimentin substantially altered the distribution of VRCs in DENV-infected cells, and the VRCs were diffused and spread throughout the cytoplasm, signifying a structural contribution of vimentin in anchoring the VRCs to the perinuclear membrane. DENV NS4A was shown to directly interact with vimentin via a specific region that lies within the first 50 amino acid residues at the cytosolic N-terminal region of NS4A [62]. Collectively, these findings suggest that DENV NS4A has a functional role in mediating the anchoring of VRCs to the perinuclear membrane, thus facilitating efficient viral RNA replication (Table 1, Figure 2).

A nuclear ribonucleoprotein polypyrimidine tract-binding protein (PTB) has been shown to be involved in pre-mRNA processing [74], polyadenylation regulation [75] and 5′ cap-independent translation of viral/cellular RNA mediated by an internal ribosome entry site [76,77,78,79]. PTB has been found to regulate viral RNA transcription, viral protein translation and viral production of a number of viruses such as picornavirus, coronavirus, herpes virus [80,81,82] and hepatitis C virus [83,84,85,86]. PTB has been reported to bind to untranslated regions of flavivirus genomes [87,88]. Interactions of PTB with the DENV RNA genome and with DENV NS4A have been identified [89], suggesting that DENV NS4A indirectly binds to DENV RNA genome by associating with PTB. Reducing PTB-DENV RNA genome binding via knockdown of PTB reduced synthesis of the minus-strand RNA intermediate which reduced DENV replication, demonstrating the biological significance of these interactions in the DENV replication cycle [89] (Figure 2).

An unprocessed NS3–NS4A has been detected as a transient intermediate during flavivirus polyprotein processing [90,91,92], and is thought to have a possible role as a protease that is responsible for *trans* cleavage at the NS4B-NS5 junction [91]. The presence of an NS3-NS4A intermediate has also led to a hypothesis that NS4A is an essential cofactor of the NS3 helicase required for unwinding of viral RNA during replication. Based on the in vitro enzymatic assay of the individual WNV NS3helicase (NS3hel), and a NS3hel fused with the cytosolic N-terminal residues 1 to 50 of NS4A (NS3hel–NS4A), the NS3hel–NS4A showed a dramatic decrease in ATPase activity, but a comparable oligonucleotide duplex unwinding activity as compared to the individual NS3hel. The results showed that while NS4A had no significant effect on the oligonucleotide duplex unwinding rate of the NS3hel, the presence of NS4A allowed the NS3hel–NS4A to conserve energy in the course of oligonucleotide duplex unwinding and enabling the NS3hel to sustain the unwinding rate of the viral RNA under ATP-deficient conditions. NS4A is therefore suggested to function as a cofactor that regulates the ATPase activity of NS3hel [64]. This findings directly complements a study showing that HCV NS4A enhanced the ability of NS3hel to bind RNA in the presence of ATP, thus acting as a cofactor for HCV NS3hel [93] (Figure 2).

NS1, another component of VRCs [17,37], has an essential but as yet unclear role in viral RNA replication, as evidenced by the findings that mutations in YFV NS1 profoundly inhibited RNA replication [18,94]. A YFV genome containing a large in-frame deletion in the NS1 gene, YFΔSK, has been found to be severely defective in accumulation of the minus-strand RNA intermediate [16], and was not complemented in *trans* by DENV NS1 [95]. However, the RNA replication defect of YFΔSK could be restored by an adaptive mutation in NS4A [95], indicating that the interaction between NS1 and NS4A is required for viral RNA replication (Figure 2). DENV NS1 has been shown to physically interact with the NS4A-2k-NS4B cleavage intermediate, but not with fully processed NS4A or NS4B, and the interaction was found to play a critical role in viral RNA replication (Figure 2). However, this interaction is not required for the role of NS1 in VP formation [96].

NS4B is a part of VRCs, as it has been found to colocalize with viral dsRNA and NS3 in the perinuclear region [70] and NS4A and NS4B have been suggested to function cooperatively in viral RNA replication based on their functional similarities. In addition to NS4A, NS4B has been shown to play an important role in cellular membrane reorganization, thus facilitating efficient viral RNA replication [44]. While WNV NS4A regulates the ATPase activity of NS3hel [64], DENV NS4B directly interacts with NS3 and enhances the overall helicase activity of NS3 by dissociating it from ssRNA and thereby enabling it to bind to a new nucleotide duplex [71]. Similar to NS4A, NS4B has been found to have a genetic interaction with NS1 to modulate viral RNA replication. The RNA replication defect of WNV containing NS1 mutations (RQ10NK) could be rescued by a F86E mutation in NS4B. A novel physical interaction between NS1 and NS4B has been demonstrated and suggested to be a mechanism by which luminal NS1 conveys signals to the cytoplasm to regulate RNA replication [97]. An interaction between NS4A and NS4B has been identified and has been demonstrated to be required for viral RNA replication. A recombinant DENV 1 bearing mutations in the N-terminal cytoplasmic portion of NS4A (in which residues 27 to 34 were replaced by the corresponding region from JEV) is defective in viral replication. The replication defect can be restored by a non-synonymous mutation in the pTMS3 of NS4B [98]. NS4A has been shown to directly interact with NS4B in DENV 2 infected cells and when co-expressed independently. The determinants for the NS4A-NS4B interaction are amino acids 40 to 76 spanning the pTMS1 (amino acids 50 to 73) of NS4A, and amino acids 84 to 146 spanning the pTMS1 (amino acids 101 to 129) of NS4B [63]. As pTMS1 of DENV 2 NS4A is required for both NS4A homo-oligomerization essential for induction of membrane curvature [55], and the NS4A–NS4B interaction [63], this may suggest that NS4A regulates the transition from VP formation to VRC formation through the switch of pTMS1 binding from NS4A to NS4B. Mutations L48A, T54A and L60A in DENV NS4A that affected the NS4A–NS4B interaction drastically reduced or abolished viral replication (Figure 1, Table 1). On the other hand, mutations F71A and G75A in NS4A that had no effect on the NS4A–NS4B interaction only slightly reduced viral replication [63]. These results suggest a biological significance of the NS4A–NS4B interaction in DENV 2 replication. 

## 3. NS4A Mediates Flavivirus Pathogenesis

### 3.1. NS4A Antagonizes the Interferon Response and Manipulates the Unfolded Protein Response

Interferon (IFN) response is a crucial innate antiviral mechanism of the host cells [99,100]. It is primarily initiated by the recognition of viral dsRNA intermediates by retinoic acid-inducible gene I (RIG-I)-like receptors (RLRs), RIG-I and Melanoma differentiation-associated gene 5 (MDA5), which are members of the DExD/H-box family of RNA helicases. The recognition leads to the conformational change of RIG-I/MDA5 in such a way that exposes its N-terminal caspase-recruitment domains (CARD), which are subsequently bound by the CARD of mitochondrial antiviral adaptor protein (MAVS). MAVS then recruits tumor necrosis factor (TNF) receptor-associated factor (TRAF) 3 and TRAF6 to its C-terminus and activates downstream signaling molecules of the RIG-I/MDA5 pathway including inhibitor of kappa-B kinase epsilon (IKKε), TANK-binding kinase 1 (TBK1) and subsequently, interferon regulatory factor 3 (IRF3), which plays an important role in stimulating the expression of type I IFN (IFN-I) [100,101,102,103]. IFN-α/β response occurs upon the binding of IFN-I to IFN-α/β receptor (IFNAR) and subsequently, through the activation of the Janus kinase/signal transducer and activator of transcription (JAK/STAT) pathway, and the transcriptional induction of a number of IFN-α/β-stimulated genes (ISGs) mediated through the IFN-α/β-stimulated response element (ISRE) [104], inducing an antiviral state. However, flaviviruses have been shown to circumvent IFN antiviral activities, and establish a successful infection in human [105], with flavivirus NS4As playing a crucial role in counteracting IFN-I production [11,12,106,107,108,109].

**Figure 3:**
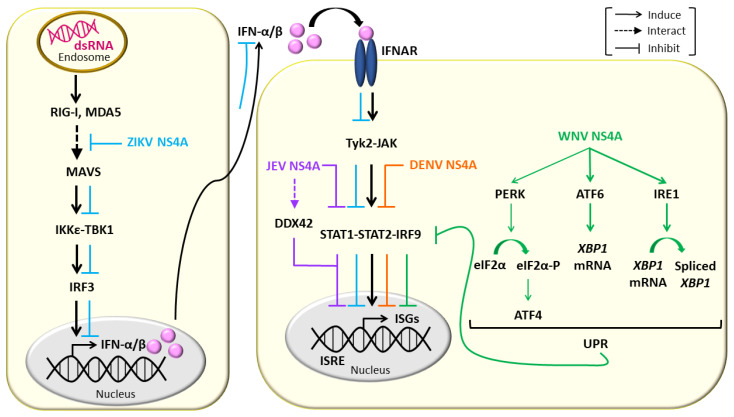
NS4As modulate the interferon response. NS4As manipulate RIG-I-like receptor signaling, DDX42 helicase, JAK/STAT signaling and the unfolded protein response (UPR) to counteract the interferon-α/β (IFN-α/β) response.

ZIKV NS4A has been demonstrated to repress RLR signaling (Figure 3), as evidenced by the finding that, when expressed independently in the presence of polyI:C (which stimulates cellular RLR signaling, thus inducing IFN-I expression), ZIKV NS4A reduced ISRE promoter activity (activated by IRF3/7 or STAT1/2) [108], and mRNA levels of IFN-stimulated genes, *ISG15* [108], *interferon induced protein with tetratricopeptide repeats 1* and *2′-5′-oligoadenylate synthetase 1* [106,108]. However, ZIKV NS4A does not interfere with Toll-like receptor (TLR) signaling as shown by that, when co-expressed with a modified TLR3 that localizes to the plasma membrane (without stimulation of RLRs by polyI:C), ZIKV NS4A affected neither the *ISRE* promoter activity nor the *IFN beta 1* and *TNF-α* mRNA level [108]. Furthermore, ZIKV NS4A has been demonstrated to suppress IFN-I induction mediated by ectopic expression of ΔRIG-I (containing only the two CARDs domain) [108], constitutively active RIG-I-1-228 [109] and MDA5 [108,109], as well as downstream signaling molecules of the RIG-I/MDA5 pathway, MAVS, IKKε, TBK1, full-length IRF3 (IRF3-FL) and regulatory domain-deleted IRF3-1-390 [109]. In addition, ZIKV NS4A has been shown to interact with the CARD domain of MAVS but not RIG-I or MDA5 [106,108]. ZIKV NS4A competed with RIG-I/MDA5 for the binding to MAVS [108], and significantly decreased the interaction between MAVS and its downstream effectors TRAF6 or TBK1 [106]. ZIKV NS4A was therefore, suggested to be a dominant negative interactor of RLR signaling, which competes with RIG-I/MDA5 for binding to the CARD of MAVS, and subsequently modulates the downstream signaling, resulting in the suppression of IFN-I production [106]. Interestingly, ZIKV NS4A has also been shown to suppress IFN-I induction upon vesicular stomatitis virus (VSV) infection and promote VSV replication in 293T cells, suggesting that the antagonistic effect of ZIKV NS4A on IFN-I production could occur in the context of actual viral infection [106].

DENV has been reported to antagonize the IFN response in humans [110], and DENV infection has also been shown to counteract the action of IFN in vitro [111]. Potential DENV-derived IFN antagonists have been identified based on the ability of each individual DENV protein to facilitate the replication of IFN-sensitive Newcastle disease virus (NDV) in human A549 cells transfected with the plasmids expressing the corresponding DENV proteins, producing IFN. NDV replication was found to be enhanced in A549 expressing DENV NS4A, NS4B and NS2A, as compared to the cell transfected with empty plasmid. DENV NS4A, NS4B and NS2A were shown to reduce the activation of *ISRE-54* promoter (stimulated by IFN-α/β through the activation of the STAT1/STAT2/ISG factor 3 transcription factor) to different extents in Vero cells after stimulation with exogenously added IFN-α/β, suggesting that these DENV proteins interfere IFN-mediated signaling pathway (Figure 3). Interestingly, co-expression of these DENV proteins in Vero cells was found to further enhance their antagonistic effects in the IFN signaling [12]. In contrast, it has been reported that heterologous expression of DENV NS4A-NS4B fusion protein in Vero cells did not block IFN signaling unless the fusion protein was processed by the co-expressed viral peptidase NS2B-NS3, indicating that the IFN-antagonist functions of DENV NS4A and NS4B required proper viral polyprotein processing [11].

JEV NS4A has been demonstrated to have an antagonistic effect on the IFN-I signaling by reducing the phosphorylation levels of STAT1 and STAT2, thus blocking the downstream activation of the JAK-STAT signaling pathway. JEV NS4A was shown to specifically interact with ATP-dependent RNA helicase DDX42. The DDX42 helicase is a member of the DExD/H-box family of RNA helicases, like RIG-I and MDA5. Overexpression of DDX42 RNA helicase increased the activation of IFN-I signaling induced by exogenously added IFN-β. These data suggest that DDX42 helicase acts as a dsRNA sensor that activates the IFN-I response upon flavivirus infection, and the binding of JEV NS4A to the DDX42 helicase could block IFN-I response [107] (Figure 3).

The unfolded protein response (UPR) is an intracellular defense mechanism that is activated in response to accumulation of unfolded or misfolded proteins in the ER occurring upon exposure to various internal or external stresses. The UPR acts to increase ER volume and ER components including chaperones required for protein folding, increase protein degradation, and inhibit protein translation to decrease protein input. There are three main branches of the UPR: the protein kinase-like ER resident kinase (PERK), the activating transcription factor 6 (ATF6) and the inositol-requiring enzyme 1 (IRE1) [112]. Flaviviruses have been found to up-regulate the UPR and manipulate downstream signaling to favor their replication [113,114,115,116]. The strongly induced UPR observed upon WNV infection was biased towards the ATF6 and IRE1 branches, as demonstrated by the strong up-regulation of *Xbp-1* expression and splicing, with a low level of PERK activation, as demonstrated by a modest increase in *ATF4* expression [113]. When expressed independently, WNV NS4A (without or with 2k) was shown to strongly induce *Xbp-1* expression and splicing, coinciding with a reduction in STAT1 nuclear trafficking, an indicator of reduced IFN signaling (Figure 3). A progressive C-terminal deletion of the hydrophobic regions of WNV NS4A resulted in a stepwise decrease in *Xbp-1* expression and restoration of STAT1 nuclear trafficking, demonstrating a correlation between the UPR and inhibition of IFN signaling [113]. These findings suggest that the hydrophobicity of WNV NS4A is essential for WNV to manipulate the UPR and to inhibit the IFN response to facilitate its replication.

### 3.2. NS4A Modulates Autophagy

**Figure 4:**
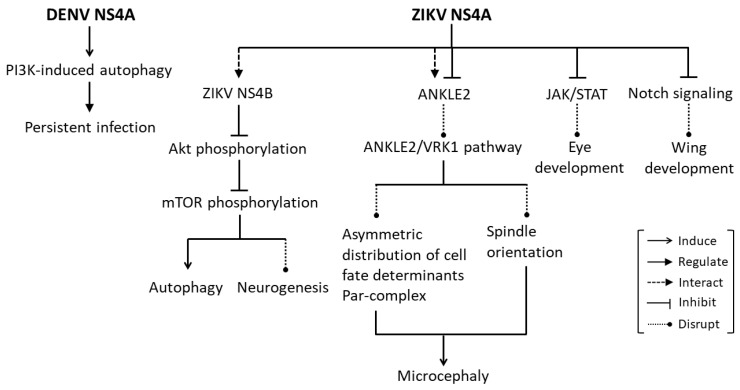
NS4As modulate developmental processes. NS4As modulate autophagy to facilitate persistent infection and manipulate a number of signaling pathways, i.e., Akt-mTOR, ANKLE2/VRK1, JAK/STAT and Notch signaling resulting in developmental defects.

Autophagy is an essential mechanism for maintaining cellular homeostasis, by which unnecessary or dysfunctional cellular components are sequestered in double-membrane vesicles (autophagosomes), which in turn fused with lysosome (autolysosome) and the contents in the autolysosome are eventually degraded and recycled. It has been commonly found that flaviviruses often persist in the liver and kidney following the acute phase of infection without cells undergoing induced cell death. Induction of autophagy has been suggested as a mechanism utilized by flaviviruses to evade the host immune response to establish a persistent infection. DENV and Modoc infection have been shown to up-regulate autophagy in MDCK renal epithelial cells and fibroblasts, and subsequently protect them from death. Inhibition of autophagy by inactivation of phosphoinositide 3-kinases (PI3K) using wortmannin or 3MA reduced protection against death conferred by DENV and Modoc virus, indicating that protection induced by these viruses is mediated by PI3K-dependent autophagy. In addition, in autophagy-deficient fibroblast cell lines, *Beclin^+/−^* and *ATG5^−/−^*, protection conferred by these two viruses was also reduced, emphasizing an important role of autophagy in flavivirus-induced protection against cell death. Inhibition of autophagy also attenuated DENV and Modoc virus in MDCK cells, indicating that autophagy enhances replication of these viruses in such cell type. When expressed independently, DENV NS4A and Modoc virus NS4A were the only viral proteins that protected MDCK cells against death in a manner similar to that of the live viruses and were also shown to induce PI3K-dependent autophagy. These findings suggest that flavivirus NS4A plays a major role in the up-regulation of PI3K-dependent autophagy induced upon flavivirus infection, which confers protection of cells against death, providing a well-protected host cell for replication of flaviviruses during their persistent infection [117] (Figure 4).

### 3.3. NS4A Causes Developmental Defects

ZIKV infection is known to cause microcephaly and other developmental defects [118,119,120]. ZIKV infection has been shown to impair growth and proliferation of induced pluripotent stem cells (iPSC), iPSC-derived neural stem cells (NSCs) and human fetal neural stem cells (fNSCs) [121,122,123]. Ectopic expression of either ZIKV NS4A or NS4B in human fNSCs inhibited neurosphere formation and reduced neurosphere size. Interestingly, co-expression of ZIKV NS4A and NS4B further reduced neurosphere formation and average neurosphere size. However, co-expression of DENV NS4A and NS4B did not show any significant impairment of neurosphere formation. Individual expression or co-expression of ZIKV NS4A and NS4B also reduced proliferation rates of fNSCs, and differentiation rates of fNSCs into neurons or astrocytes. Furthermore, ZIKV infection was shown to induce autophagy in fNSCs, which in turn promotes ZIKV replication. Individual expression of either ZIKV NS4A or NS4B showed subtle effects on autophagy induction, whereas co-expression of these two ZIKV proteins resulted in a significant up-regulation of autophagy. ZIKV NS4A was further shown to interact with NS4B in cells, suggesting that ZIKV NS4A and NS4B function cooperatively to induce efficient autophagy upon ZIKV infection [122]. Akt-mTOR signaling is essential for neurogenesis by fNSCs and for the induction of autophagy [124]. Akt, a central signaling molecule in the PI3K pathway upstream of mTOR, plays crucial roles in brain development [125], and non-functional Akt mutation leads to microcephaly [126]. Inhibition of mTOR in the developing brain also causes microcephaly, and inactivation of mTOR by AMPK and p53 signaling induces autophagy [127,128,129]. Individual expression of either ZIKV NS4A or NS4B in fNSCs suppressed Thr308 and Ser437 phosphorylations of Akt, whereas co-expression of these two ZIKV proteins intensified the suppressing effects and consequently led to reduced levels of mTOR phosphorylation at Ser2448. Overexpression of the constitutively active form of Akt3 (myr-HA-Akt3 E17K) in fNSCs was shown to down-regulate autophagy induced by ZIKV infection or NS4A-NS4B co-expression. Collective these findings suggest that ZIKV NS4A and NS4B impair the neurogenesis of fNSCs and increase autophagy through inhibition of the Akt-mTOR signaling pathway [122] (Figure 4). However, these results seem to be in contradiction with a study demonstrating that ZIKV infection in neuronal and glia cells activated the mTOR complex (mTORC) 1 and mTORC2, which subsequently suppressed autophagy, resulting in viral protein accumulation and progeny virus production [130]. The contradictory findings as to the roles of mTOR and autophagy in ZIKV infection could be a consequence of different cell types, experimental model systems or the temporality of the events being evaluated. Moreover, the different molecular tools used to study mTOR signaling whether phosphorylation status of mTOR at S2448 or of mTOR substrates, p70S6K, ULK1, and Akt might account for the conflicting findings [130]. 

*Ankyrin repeat and LEM domain containing 2* (*ANKLE2*) has been shown to be associated with hereditary microcephaly, as mutations in *ANKLE2* causes microcephaly in humans [131,132] and *Drosophila* [133]. The functions of ANKLE2 have also been shown to be evolutionarily conserved from *Drosophila* to human, as expression of human ANKLE2 in *Drosophila Ankle2* heterozygous hypomorphic mutants (*Ankle2^A^*) rescues the phenotype [132]. ZIKV NS4A has been found to physically interact with ANKLE2 in human cells, and ectopic expression of ZIKV NS4A in *Drosophila* larval brain resulted in microcephaly, increased apoptosis, and reduced proliferation of neuroblasts. In comparison with ZIKV NS4A, DENV 2 NS4A was shown to interact with ANKLE2 with a lower affinity, without significantly inducing microcephaly, consistent with the fact that DENV does not cause microcephaly in human. Ectopic expression of ZIKV NS4A in *Drosophila Ankle2^A^* mutants led to a more severe microcephaly phenotype. The microcephaly phenotype caused by ectopic expression of ZIKV NS4A was found to be rescued by ectopic expression of human ANKLE2. These data suggest that ZIKV NS4A interacts with the ANKLE2 protein and inhibits ANKLE2 function, thus contributing to ZIKV-induced microcephaly [134] (Figure 4).

ANKLE2 has been found to be localized to the ER and nuclear envelope, similar to ZIKV NS4A. Disruption of *Ankle2* resulted in an aberrant nuclear envelope and ER distribution, leading to the release of a protein kinase Ballchen (ball; *Drosophila* homolog) or Vaccina-Related Kinase 1 (VRK1; human homolog) into the cytosol of fly neuroblasts and human primary fibroblasts, respectively. This was found to be associated with abnormal localization of Par-complex, i.e., atypical protein kinase C (aPKC), Par-6, Bazooka (Baz), and Miranda (Mira), which are required for establishing polarity during asymmetric division of neuroblasts in *Drosophila*, as well as with spindle orientation defects and reduced aPKC phosphorylation. Removal of one copy of *ball* or *lethal(2) giant larvae* (*l(2)gl*) in the *Ankle2^A^* mutant rescued the microcephaly phenotype, suggesting that function of ANKLE2 is modulated by aPKC and l(2)gl levels. Similar to the *Ankle2^A^* mutant, ectopic expression of ZIKV NS4A in *Drosophila* neuroblasts not only caused microcephaly [134] but also resulted in an aberrant apical aPKC localization, Mira domain expansion and spindle orientation defects. These phenotypes induced by ectopic expression of ZIKV NS4A were rescued by removing a single copy of *ball* or *l(2)gl*. These findings suggest that ZIKV-induced microcephaly is mediated by ZIKV NS4A which hijacks the ANKLE2-ball (VRK1) pathway and affects asymmetric distribution of cell fate determinants, resulting in neuroblast division and brain development defects [135] (Figure 4).

Apart from acting as a key regulator of IFN signaling, the JAK/STAT pathway has also been demonstrated to have a pleiotropic function in regulating tissue development [136]. ZIKV infection in *Drosophila* has been shown to induce up-regulation of negative regulators of JAK/STAT signaling, E(bx) and suppressor of cytokine signaling 36E (Socs36E). Eye-specific overexpression of NS4A resulted in a significant reduction in the developing eye size [137], a phenotype also observed as a consequence of loss of function of the *hopscotch* (*hop*) gene (encoding JAK) [138,139]. The reduced eye size caused by ZIKV NS4A overexpression correlated with the reduction in expression levels of the targets of JAK/STAT signaling, *chinmo*, *Mo25* and *domeless*, and was linked with a reduced rate of cell proliferation in the eye imaginal epithelia, although the rate of apoptosis remained unaffected. Overexpression of ZIKV NS4A together with the dominant-negative form of domeless, or in combination with STAT1 knockdown, resulted in a synergistic reduction in eye size, while co-expression of ZIKV NS4A with activated Hop kinase partially rescued the eye overgrowth. These data demonstrate the interaction between ZIKV NS4A and JAK/STAT signaling components [137]. Apart from regulating eye development, JAK/STAT signaling has also been shown to regulate wing development [140,141]. Wing-specific overexpression of ZIKV NS4A resulted in thickening of the wing vein, a phenotype characteristic also found upon overexpression of Socs36E (a negative regulator of JAK/STAT signaling), and mutation in Notch signaling [137]. ZIKV NS4A overexpression was shown to reduce expression of Notch protein as well as Wg and Cut [137], which are targets of Notch signaling [142,143]. Collectively, these findings suggest that ZIKV NS4A mediates ZIKV-induced restricted eye and wing growth, through downregulation of JAK/STAT and Notch signaling, respectively [137] (Figure 4).

## 4. Conclusions

NS4A is one of the flavivirus NS proteins that remains poorly characterized. Apart from being known for its role in mediating flavivirus-induced cellular membrane remodeling [41,44], NS4A also act as an essential component of VRCs, that physically interacts with host factors or other flavivirus NS proteins to promote viral replication [62,63,89,95,98] (Figure 1 and Figure 2, Table 1). Interestingly, unprocessed intermediates containing NS4As also have critical roles in viral RNA replication [64,96] (Figure 2). Importantly, NS4A contributes to the pathogenesis of flaviviruses by counteracting the IFN response, modulating the UPR and autophagy, as well as causing developmental defects, through hijacking of a number of cellular signaling pathways [11,12,106,107,108,109,113,117,122,134,135,137] (Figure 3 and Figure 4). These highlight NS4A as a highly promising antiviral drug target. However, more studies are required to gain further insights into the roles of NS4A in flavivirus infection, as its critical role in a number of processes suggests that this protein and its interaction may be a good candidate for the development of effective antivirals.
